# Pegcetacoplan in idiopathic and familial pediatric C3 glomerulopathy

**DOI:** 10.1007/s00467-025-07092-7

**Published:** 2025-12-08

**Authors:** Elena Román Ortiz, Marina Sáez Bello, Andrea Reparaz Suevos, Marisa Perez Ebri, Francisco Aguilar Bacallado, Pilar LLopis Salvia, Mónica Climente Martí, Santiago Rodriguez de Córdoba

**Affiliations:** 1 Unidad de Nefrología Pediátrica, Hospital Universitario Dr. Peset, Valencia, Spain; 2 Servicio de Farmacia, Hospital Universitario Dr. Peset, Valencia, Spain; 3 Laboratorio de Complemento, Centro de Investigaciones Biológicas-Margarita Salas, Madrid, Spain; 4 Servicio de Anatomía Patológica, Hospital Universitario Dr. Peset, Valencia, Spain; 5 Servicio de Pediatría, Hospital General Universitario de Valencia, Valencia, Spain; 6 Fundació per al Foment de la Investigació Sanitària I Biomèdica de La Comunitat Valenciana (FISABIO), Valencia, Spain

**Keywords:** C3 glomerulopathy, C3 glomerulonephritis, C3 inhibitor, Pegcetacoplan, Complement, Children

## Abstract

**Background:**

C3 glomerulopathy (C3G) and immune complex-mediated membranoproliferative glomerulonephritis (IC-MPGN) represent a continuous spectrum of a glomerular disease driven by dysregulation of the complement and characterized by C3 deposition alone or associated with immunoglobulins. Despite the significant burden and poor prognosis associated with these conditions, no therapies have been approved for their treatment in children.

**Methods:**

In this observational study, we present three pediatric cases that span the clinical and pathogenic spectrum of C3G/IC-MPGN, including multi-resistant nephrotic syndrome with clear terminal pathway activation, familial genetic C3G with early anti-C3 intervention, and multi-resistant nephrotic syndrome of unclear etiology, likely related to IC.

**Results:**

All three patients were successfully treated with the C3/C3b inhibitor pegcetacoplan, that inhibited C3, blocked C3 consumption, and restored physiological C3 levels, leading to significant proteinuria reduction within the first month. After six months, patients experienced notable improvements in kidney function, a complete remission of nephrotic syndrome, and normalized proteinuria levels. There were no treatment-related adverse events, only mild infections that resolved with standard oral therapy.

**Conclusions:**

These findings support the potential of C3 inhibition with pegcetacoplan in pediatric patients with refractory or genetic C3G.

**Graphical abstract:**

A higher resolution version of the Graphical abstract is available as Supplementary information.

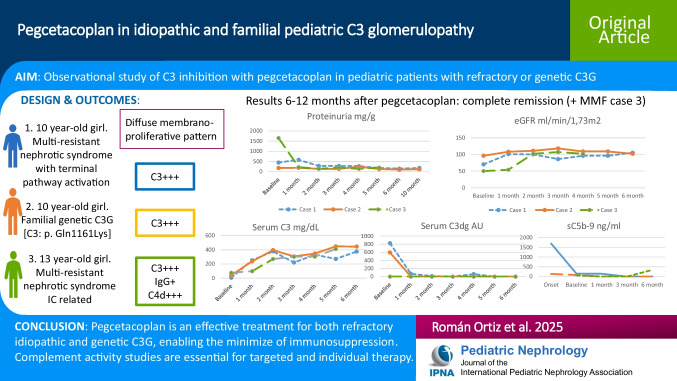

**Supplementary Information:**

The online version contains supplementary material available at 10.1007/s00467-025-07092-7.

## Introduction

C3 glomerulopathy (C3G) and immune complex-mediated membranoproliferative glomerulonephritis (IC-MPGN) are heterogeneous kidney diseases characterized by overactivation of the alternative complement pathway associated with pathogenic variants in complement genes or autoantibodies against its components, such as factor H, C3, and C3/C5 convertases (C3/C5 nephritic factors or C3/C5Nefs) [1, 2]. The resulting hyperactivation leads to the deposition of C3 breakdown fragments in the glomerulus, causing inflammation and progressive kidney damage in two main histological forms: dense deposit disease (DDD) and C3 glomerulonephritis (C3GN) [3, 4]. The prognosis is poor with nearly 70% of children and 40–50% of adults patients reaching kidney failure within 10 years after diagnosis [5]. In children, recommended treatment approaches are based on retrospective studies and expert opinion. Management typically includes renin–angiotensin–aldosterone system (RAAS) blockers and immunosuppressants like steroids, mycophenolate mofetil, cyclophosphamide, calcineurin inhibitors, and rituximab [6].

Given the role of complement dysregulation in the pathogenesis of C3G, eculizumab was the first complement inhibitor investigated but reported results have been highly variable, likely because activation of the terminal complement pathway is not a determining factor in many cases [5, 6].

Pegcetacoplan is a C3/C3b inhibitor approved by the European Medicines Agency for Paroxysmal Nocturnal Haemoglobinuria (PNH) [7]. At present, pegcetacoplan is being investigated in phase III trials for the treatment of adult and adolescent patients with C3G or primary immune complex-mediated membranoproliferative glomerulonephritis (IC-MPGN) [8].

Previous data from Phase 2 studies reported a significant reduction in proteinuria in patients treated with pegcetacoplan with native and recurrent post-transplant C3G/IC-MPGN [9, 10]. A phase 3 study included 55 adolescents with C3G or IC-MPGN, experiencing a proteinuria reduction of 74.5% [11]. Furthermore, pegcetacoplan was effective in an additional five children with C3G treated for 12 weeks, with an observed reduction in proteinuria to less than 30% from baseline [12].

In the current observational study, we discuss three pediatric patients with C3G treated with pegcetacoplan: two with multi-resistant nephrotic syndrome and one with genetic familial C3G. These cases add to the growing evidence of pegcetacoplan’s potential in managing refractory or genetic C3G in children.

## Methods

Between 2020 and 2024, six patients with C3G were identified in our unit. Of these, three achieved complete remission with corticosteroid treatment. The characteristics of the three patients from this study are described in Table 1 and Supplementary Tables S1 and S2. Genetic and functional complement studies were done at diagnosis and included next-generation sequencing (NGS) screening of all complement genes, with assessment for copy number variation and rearrangements in the CFH-CFHRs region, and anti-factor H (FH), and anti-C3 convertase (C3bBb, C3Nef) autoantibodies (Supplementary Table S2). C3 activation was monitored by measuring C3 and its activated fragments (iC3b and C3dg) plasma levels, while terminal pathway activation was assessed by determining levels of soluble C5b-9 complex (sC5b-9), both before and after initiation of treatment.

**Table 1:** Patient characteristics of the children with C3G treated with pegcetacoplan

	CASE 1	CASE 2	CASE 3
Age at diagnosis (year)	7	8	11
Clinical presentation	Nephrotic syndrome, macrohematuria, Hypertension, GFR < 85	Proteinuria GFR < 85	Nephrotic syndrome, microhematuria, Hypertension, GFR < 85
Laboratory at diagnosis			
eGFR CkiD* ml/min/1.73 m^2^	70	76	74
Proteinuria g/24 h	4	0.580	6.7
Albumin g/dl	2.5	3.6	2.1
C3 mg/dl	4	50	75
Time to C3G diagnosis	1 month	6 months	2 month IC-MPGN 24 months C3G
Histological diagnosis	C3G	C3G	IC-MPGN switches to C3G
Previous treatment	Prednisone,candesartan, MMF, tacrolimus, eculizumab, ravulizumab	Candesartan	Prednisone, losartan, MMF, tacrolimus
Time of previous treatment to pegcetacoplan	20 months	10 months	24 months
Follow-up months - Diagnosis - Pegcetacoplan	40 12	22 12	29 6
Current status	Complete remission with pegcetacoplan Normal GFR	Complete remission with pegcetacoplan Normal GFR	Complete remission with pegcetacoplan + MMF Normal GFR

^*^eGFR = 39.8 × [ht/Scr]^*0.456*^ × [1.8/cysC]^*0.418*^ × [30/BUN]^*0.079*^ × [1.076^*male*^] [1.00^*female*^] × [ht/1.4]^*0.179*^

*GFR* glomerular filtration rate, *C3G* C3 glomerulonephritis, *IC MPGN* immunocomplex membranoprolipherative glomerulonephritis, *MMF* mycophenolate mofetil

After a disease progression period ranging from 10 to 29 months, pegcetacoplan was requested for off-label use following its approval for the treatment of PNH. Dosing was based on the regimen used in the phase 3 VALIANT trial [13] (Table 2). Families were trained in the administration procedure. The first two doses were administered in the hospital, with subsequent doses given at home. Currently, all three patients continue to receive treatment with pegcetacoplan, combined with RAAS blockade, and mycophenolate mofetil in one case. All patients underwent comprehensive immunization against Hemophilus influenzae, pneumococcus, meningococcus (ACWY, B), influenza and COVID-19.

**Table 2:** Pegcetacoplan dosage in pediatric patients

Weight	1 st dose (mg) (infusion volume in ml)	2nd dose (mg) (infusion volume in ml)	Maintenance dose (mg) (infusion volume in ml)
Adults and adolescents ≥ 50 kg	1.080 (20)	1.080 (20)	1.080 twice weekly (20)
Adolescents between 35 and < 50 kg	648 (12)	810 (15)	810 twice weekly (15)
Adolescents between 30 and < 35 kg	540 (10)	540 (10)	648 twice weekly (12)

Source: Vivarelli et al. [13]

## Results

### Case 1

**Fig. 1:**
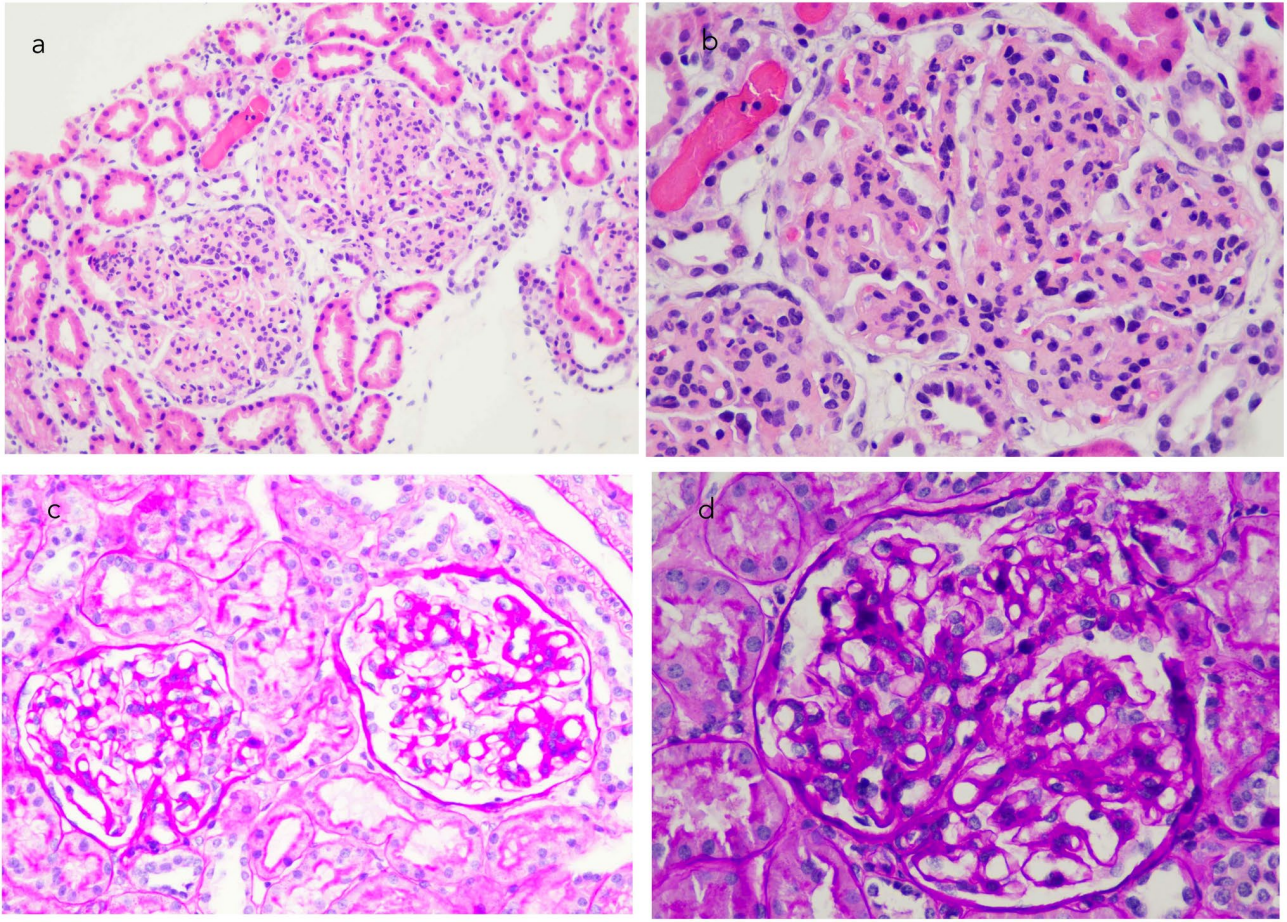
Kidney biopsy. **a**) Case 1: Glomeruli with accentuation of lobularity and increased cellularity with a membranoproliferative pattern (H-E 200×). **b**) Case 1: Increased mesangial matrix and cellularity, double contours, and focal infiltration of the tuft by polymorphonuclear cells (H-E 400×). **c**) Case 2: Mesangial proliferation with segmental thickening of the glomerular basement membrane (PAS 200×). **d**) Case 2: Membranoproliferative pattern with synechiae to Bowman’s capsule (PAS 400×)

**Fig. 2:**
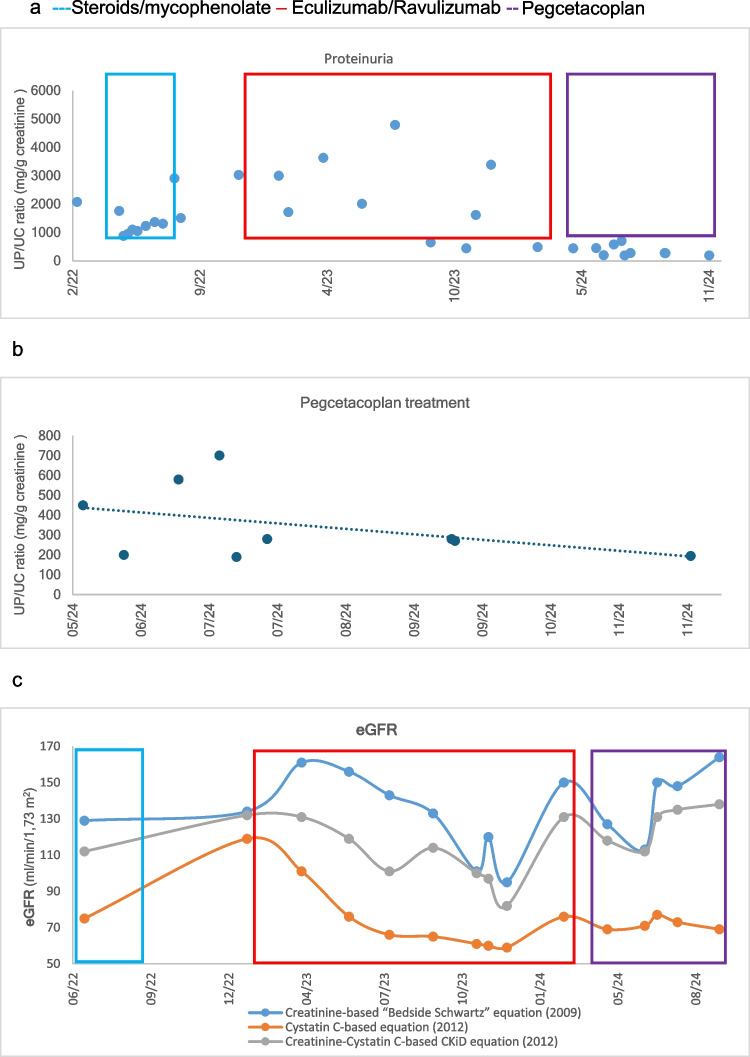
Case 1: Progression and treatment. **a**) Proteinuria/creatinine ratio (mg/g creatinine). **b**) Proteinuria/creatinine ratio (mg/g creatinine) with pegcetacoplan. **c**) Estimated glomerular filtration rate (ml/min/1.73 m^2^) calculated using the National Kidney Foundation platform available at: https://www.kidney.org/professionals/kdoqi/gfr_calculatorPed

A 10-year-old girl presented at the age of 7 with steroid-resistant nephrotic syndrome, hematuria, proteinuria, hypertension (HTN), and C3 hypocomplementemia (Table 1). A kidney biopsy confirmed C3G diagnosis with a diffuse membranoproliferative pattern and subendothelial and mesangial C3 deposits (Supplementary Table S1, Fig. 1 a, b). Initial treatment included prednisone, mycophenolate mofetil (MMF), and candesartan. Complement studies did not identify any pathogenic variants in complement genes or autoantibodies that would suggest a predisposition to C3G (Supplementary Table S2). Despite 6 months of treatment with prednisone and MMF, the nephrotic syndrome, hypertension, undetectable serum C3, and elevated sC5b-9 levels persisted. This led to the decision to initiate treatment with eculizumab (Fig. 2a, c). After 2.5 months of eculizumab treatment, the patient achieved a 75% reduction in proteinuria, partial remission of nephrotic syndrome, improved kidney function and HTN, and a decrease in sC5b-9 from 1697 to 627 UA/ml. However, C3 levels remained undetectable, and elevated levels of C3dg persisted (Table 3). Eculizumab was discontinued, and the patient continued prednisone (10 mg/d) and MMF (1200 mg/m^2^/day).

**Table 3:**
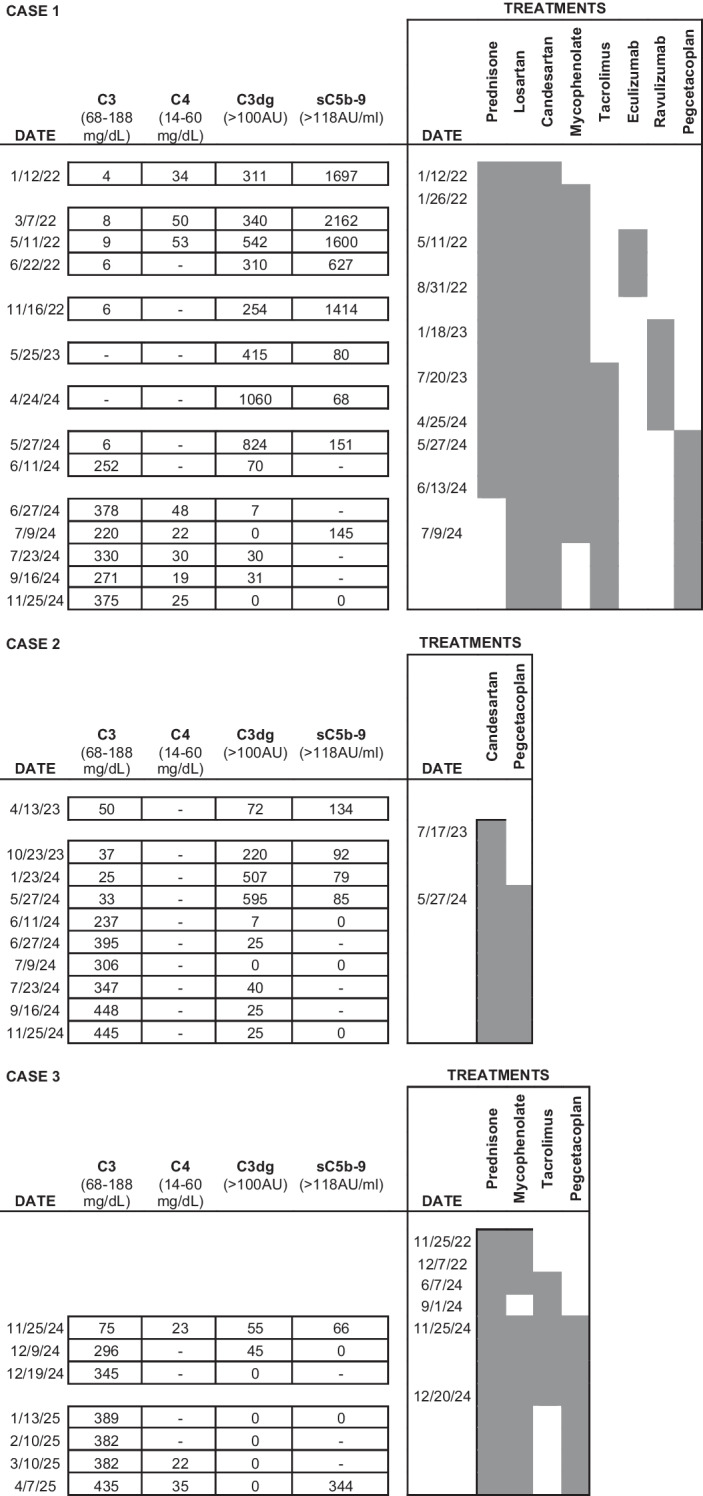
Evolution of C3 and C4 levels and complement activity with treatments

Five months later, the patient experienced a nephrotic syndrome relapse, presenting with hypoalbuminemia, severe proteinuria (urinary protein-creatinine ratio (PCR): 3 mg/g), hypercholesterolemia, and oliguria, associated with an increase in sC5b-9 levels (1414 UA/ml). As a result, anti-complement treatment with ravulizumab was restarted, and tacrolimus (Tac) was added. The patient responded with partial remission, achieving an 85% reduction in proteinuria, stabilization of the glomerular filtration rate, but no changes in complement consumption. Ravulizumab treatment was maintained for 15 months (Fig. 2a, c).

At 29 months of follow-up and 33 days after the last dose of ravulizumab, treatment with pegcetacoplan 1080 mg twice a week was initiated. This was combined with prednisone (5 mg every 48 h), mycophenolate (1250 mg/day), tacrolimus (4 mg/day), candesartan (16 mg/day), and hydrochlorothiazide (12.5 mg/day). The switch from ravulizumab to pegcetacoplan was timed halfway through the ravulizumab dosing interval to minimize the risk of relapse due to incomplete terminal pathway inhibition while switching between complement inhibitors. Antibiotic prophylaxis was maintained for two months after pegcetacoplan administration.

**Fig. 3:**
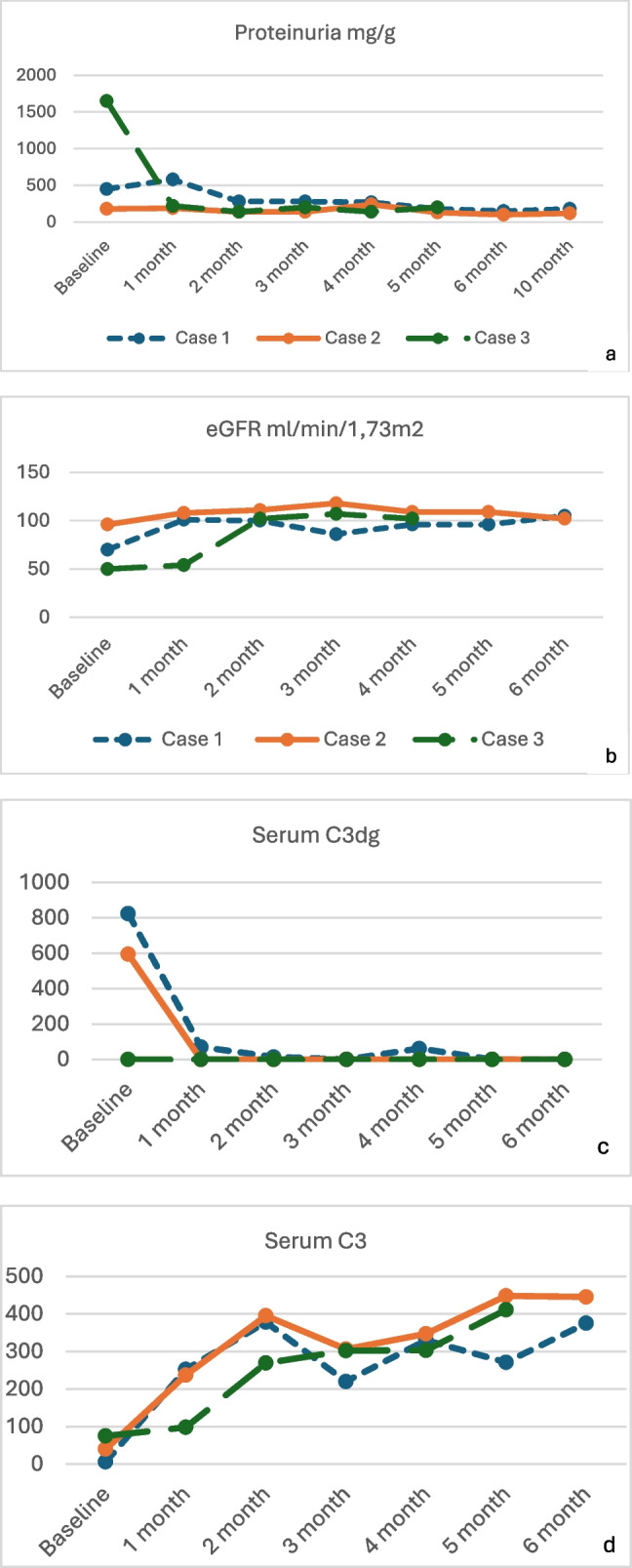
Complement activity and proteinuria before and after pegcetacoplan. **a**) Proteinuria/creatinine ratio (mg/g creatinine). **b**) Estimated glomerular filtration rate (ml/min/1.73 m^2^) calculated using the Creatinine-Cystatin C-based CKiD Eq. 2012 via the National Kidney Foundation platform available at: https://www.kidney.org/professionals/kdoqi/gfr_calculatorPed. **c**) C3 degradation products. **d**) Serum C3 levels (mg/dl)

After one month of pegcetacoplan treatment, there was a significant increase in C3 levels (from 6 to 252 mg/dl), negative serum C3dg (Table 3), an increase in estimated glomerular filtration rate (eGFR) of 30 ml/min, and a reduction in proteinuria by 37.7% and 60% at 3 and 6 months, respectively. By the fifth month of treatment, the PCR had decreased to below 200 mg/g (Figs. 2a, b, c and Fig. 3). Prednisone, mycophenolate and hydrochlorothiazide were subsequently discontinued, while remission of nephrotic syndrome, normal eGFR, a PCR of 110 mg/g, and good control of hypertension with candesartan were maintained at 12 months of follow-up.

Regarding treatment safety, the patient experienced a mild local reaction at the infusion site, which resolved with premedication using desloratadine and paracetamol during the first 3 months. On day 43 of treatment, the patient developed otitis media concomitant with mycoplasma pneumonia and adenovirus with mild right pleural effusion. This condition improved with outpatient treatment using ciprofloxacin drops, cefuroxime, and oral azithromycin. Mycophenolate was discontinued, and the pegcetacoplan dose was delayed 24 h.

### Case 2

A 10-year-old female patient was diagnosed with C3G at the age of 9 due to familial and genetic predisposition. Her father exhibited proteinuria and hypocomplementemia at age 14 and was diagnosed with C3G at age 36. He underwent treatment with prednisone, MMF, avacopan, and ultimately iptacopan. Genetic analysis identified a heterozygous variant in the C3 gene (c.3481C > A; p. Gln1161Lys), inherited from his asymptomatic mother. This variant has been characterized as a pathogenic gain-of-function mutation associated with C3G and atypical hemolytic uremic syndrome, affecting the regulation of C3 by membrane cofactor protein and FH [14]. The familial genetic study confirmed that the girl and her brother (with hemolytic uremic syndrome at age of 18 month), were also carriers of the c.3481C > A; p. Gln1161Lys mutation inherited from their father (Supplementary Table S2). The anti-FH autoantibodies and C3NeF were negative. Screening evaluation indicated asymptomatic proteinuria and hypocomplementemia with reduced C3 levels (Table 1). The patient was normotensive, with an eGFR of 76 ml/min/1.73 m^2^, and normal levels of hemoglobin, platelets, haptoglobin, and lactate dehydrogenase (LDH). Treatment commenced with a low dose of candesartan (4 mg/24 h), leading to a reduction in albuminuria to 46 mg/g. Kidney biopsy was not deemed necessary at this juncture, with close monitoring including analysis of C3 levels, sC5b-9, and C3dg.

At six months, a progressive decline in C3 levels and a marked increase in serum levels of C3 activated fragments (C3dg) were observed, indicating significant C3 overactivation (Table 3). A percutaneous kidney biopsy was conducted and revealed diffuse membranoproliferative glomerulonephritis with predominantly mesangial C3 deposits, no dense deposits on electron microscopy, and no signs of thrombotic microangiopathy (Supplementary Table S1, Fig. 1 c, d).

Treatment with pegcetacoplan was initiated at a weight-adjusted dose twice weekly, administered subcutaneously at a single infusion site. At four weeks, C3 levels increased from 40 to 237 mg/dL, with serum C3dg levels becoming undetectable (Table 3). Proteinuria decreased by 22% and 44% at 3 and 6 months respectively, achieving PCR values below 200 mg/g at three months. After 12 months of treatment, the patient maintained normal kidney function, PCR 150 mg/g, C3 levels at 140% of the upper limit of normal, and undetectable C3dg (Fig. 3).

Regarding treatment safety, after 52 days, the patient experienced a mild, non-specific episode of gastroenteritis, which was resolved within 48 h. This resulted in a 24-h delay in the administration of pegcetacoplan, with no further complications reported.

### Case 3

**Fig. 4:**
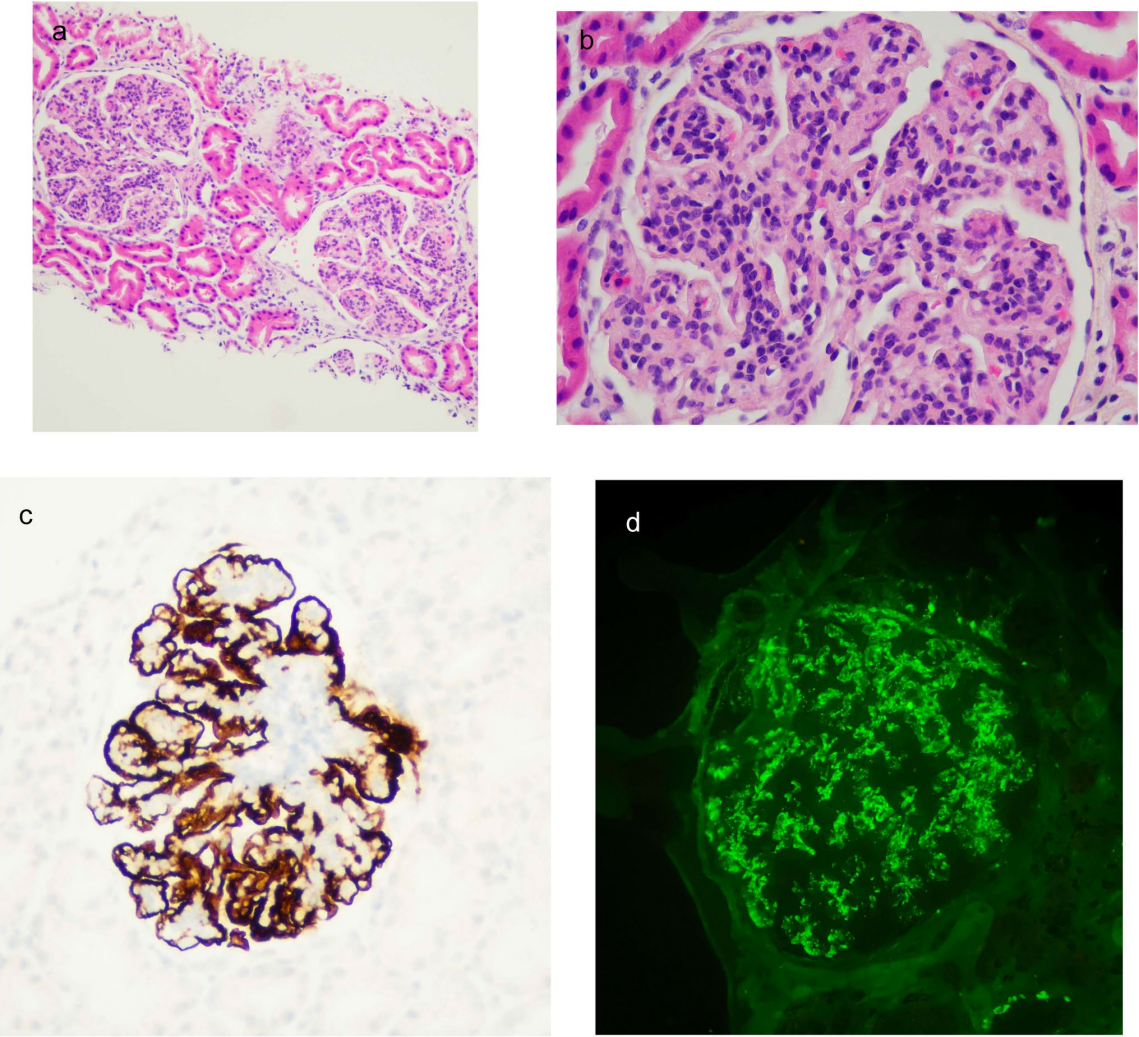
Case 3: Kidney biopsy. **a**) Large, hypercellular, lobulated glomeruli with partial compromise of capillary lumina (H-E 200×). **b**) Membranoproliferative pattern with a marked increase in mesangial and endocapillary cellularity, along with diffuse thickening of the basement membranes (H-E 400×). **c**) C4d immunohistochemistry: Intense expression in the glomerular capillary wall (C4d 400×). **d**) Immunofluorescence: Intense granular deposits in the glomerular capillary wall and mesangium of C3 (IFD C3 400×)

A 13-year-old-female adolescent presented at the age of 11 with steroid-resistant nephrotic syndrome, hypertension, an eGFR of 74 ml/min/1.73 m^2^, and C3 levels at the lower limit of the normal range (Table 1). Kidney biopsy revealed diffuse membranoproliferative glomerulonephritis with immunoglobulin G (IgG) 3 + and C3 3 + deposits, consistent with IC-MPGN. Infectious, autoimmune, and monoclonal gammopathy etiologies were ruled out. Genetic testing excluded alterations in complement-related genes, collagen IV, and podocyte genes. She was maintained on corticosteroids combined with MMF and RAAS blockade. During the first six months of treatment, kidney function remained stable, and proteinuria improved. However, at eight months, the patient experienced a relapse of nephrotic syndrome and worsening kidney function, prompting the addition of tacrolimus. After 24 months of disease progression, due to persistent multi-resistant nephrotic syndrome, a second kidney biopsy was performed, and complement studies were expanded. The second biopsy revealed severe progression of the membranoproliferative pattern with dominant C3 deposits, consistent with C3G (Supplementary Table S1, Fig. 4). The complement study showed normal C3 and C4 levels, absence of activated C3dg, C3Nef, or anti-FH autoantibodies (Table 3, Supplementary Table S2). Interestingly, the patient exhibited reduced factor I levels.

Treatment with pegcetacoplan was initiated at a dose of 1080 mg twice weekly, administered subcutaneously at a single injection site. At four weeks of pegcetacoplan treatment, C3 levels increased from 75 to 296 mg/dl, with negative serum C3dg levels and factor I levels normalized. Prednisone dose was reduced to 5 mg every 48 h and tacrolimus was discontinued, with the patient remaining on MMF (Table 1). At two months, eGFR normalized, and proteinuria decreased by 87% achieving PCR values below 200 mg/g at three months (Fig. 3). After six months of treatment, the patient maintained normal kidney function, a PCR of 200 mg/g (91% reduction in proteinuria), C3 levels at 250% of the upper limit of normal, and negative activated C3 fragments (Table 3, Fig. 3).

## Discussion

C3G poses a significant therapeutic challenge in children with no response to conventional treatment with steroids and immunosuppression [15]. Complement-targeted therapy is currently recommended for patients with aggressive presentation [16, 17].

This is the first report describing the use of pegcetacoplan in pediatric patients, correlating clinical response with serum markers of complement pathway activity before and after treatment. Our findings support the effectiveness of pegcetacoplan across different clinical scenarios, including multi-resistant C3G with terminal pathway activation, early therapy in genetic C3G, and idiopathic IC-MPGN transitioning to C3G.

However, these observations also highlight an important clinical dilemma regarding the initiation of complement inhibition in patients who exhibit biochemical evidence of complement activation despite preserved kidney function, normal serum albumin, and low-grade proteinuria controlled with ARB therapy. In our Case 2, the decision to initiate therapy was guided by the progressive deterioration of complement markers and biopsy findings, in the context of a pathogenic C3 variant and significant family history. Nevertheless, such interventions should be approached with caution until further adequately powered studies clarify the benefits and long-term implications of complement inhibition in this specific subgroup of patients.

The conditions associated with poor therapeutic responses and prognosis in C3G (NS, hypoalbuminemia, persistent hypocomplementemia, macroscopic hematuria and low glomerular filtration rate), were present in these patients [15–20].

In Case 1 eculizumab followed by ravulizumab, was administered due to the refractory disease progression, elevated sC5b-9 levels, and lack of therapeutic alternatives, as the use of C3-inhibitors was not available at that time. C5 inhibitors improved proteinuria and stabilized kidney function, consistent with the findings of Le Quintrec et al. [17], but had no effect on C3 overactivation, as evidenced by the persistent C3 consumption (Table 3). After eculizumab discontinuation, the patient experienced a relapse, prompting re-initiation of ravulizumab (Fig. 2). Discontinuation of ravulizumab followed by pegcetacoplan treatment prevented a rebound effect and blocked reactivation of the terminal pathway, suggesting effective control of the terminal pathway by C3 inactivation.

Case 2 highlights that despite proteinuria being essential for nephrologists to assess treatment necessity and response, comprehensive complement activity studies provide superior diagnosis and treatment monitoring. The patient had a clear dysregulation of the complement pathway with a genetic origin, the correlation between C3dg levels and histological damage was clear, yet kidney function was preserved, and albuminuria was mild, normalized with candesartan. Early diagnosis enabled the initiation of targeted anti-C3 therapy as a first-line treatment avoiding the side effects of steroids and conventional immunosuppression, which had already proven ineffective in her father, who carried the same pathogenic variant.

In Case 3, a transition from an initial diagnosis of IC-MPGN to C3G was documented. The etiology remains unclear, with no identified genetic or acquired factors and normal C3dg or sC5b-9 levels prior to treatment with pegcetacoplan. Interestingly, FI levels normalized within one month of initiating pegcetacoplan, leading to resolution of the nephrotic syndrome, normalization of kidney function, and remission of proteinuria within five months. These findings underscore the pivotal role of C3 activation in the pathogenesis of the disease. Microglomerular mechanisms underlying C3G may evade detection through plasma complement activation markers in some patients, as suggested by the variability in C3 hypocomplementemia and plasma sC5b-9 levels (87% and 68%, respectively, in the study by Cappoli A et al. [16]).

Across all three cases, regardless of the disease's genetic or idiopathic origin, pegcetacoplan effectively controlled C3 overactivation by halting the production of C3 downstream effectors, normalizing plasma C3 levels, and preventing sC5b-9 activation. This resulted in significant proteinuria reduction (40–90%), remission of microhematuria and preserved kidney function reducing reliance on immunosuppressive therapy.

Adverse events were mild, managed on an outpatient basis, resolved with conventional oral treatments and were classified as having "doubtful" causality according to the Naranjo algorithm [21].

While acknowledging the limitations of a single-center, three-case series, this study provides valuable evidence supporting the efficacy of C3-targeted therapy with pegcetacoplan in patients with C3G of varying etiologies. It underscores the potential of pegcetacoplan as a specific therapy for patients with ongoing complement activation like genetic C3G or those resistant to conventional treatments. Despite the promising results, experience with pegcetacoplan in pediatric C3G remains limited. Further research is needed to address unresolved questions about complement dysregulation, optimal treatment duration, and the risk of relapse or subclinical damage following treatment discontinuation.

In conclusion, these preliminary findings suggest that pegcetacoplan may be an effective treatment for both idiopathic and genetic C3G, potentially allowing for minimization or avoidance of immunosuppressive therapy. Comprehensive complement activity studies and ongoing monitoring remain essential for accurate diagnosis and individualized management in pediatric C3G patients.

## Supplementary Information

Below is the link to the electronic supplementary material.

Supplementary file1 Graphical abstract (PPTX 99 KB)

Supplementary file2 (DOCX 21 KB)

## Data Availability

All data generated or analyzed during this study are included in this published article and its supplementary information files. Additional information supporting the findings of this study are available from the corresponding author upon reasonable request.
