# Supplementary material for: Pegcetacoplan in idiopathic and familial pediatric C3 glomerulopathy
Source: Pediatr Nephrol. 2025 Dec 8;41(5):1351–60. doi: 10.1007/s00467-025-07092-7 (PMC13009099; doi:10.1007/s00467-025-07092-7)
Supplement: Supplementary file 1 — Supplementary file1 Graphical abstract (PPTX 99 KB) [file 467_2025_7092_MOESM1_ESM.pptx]

## Slide 1
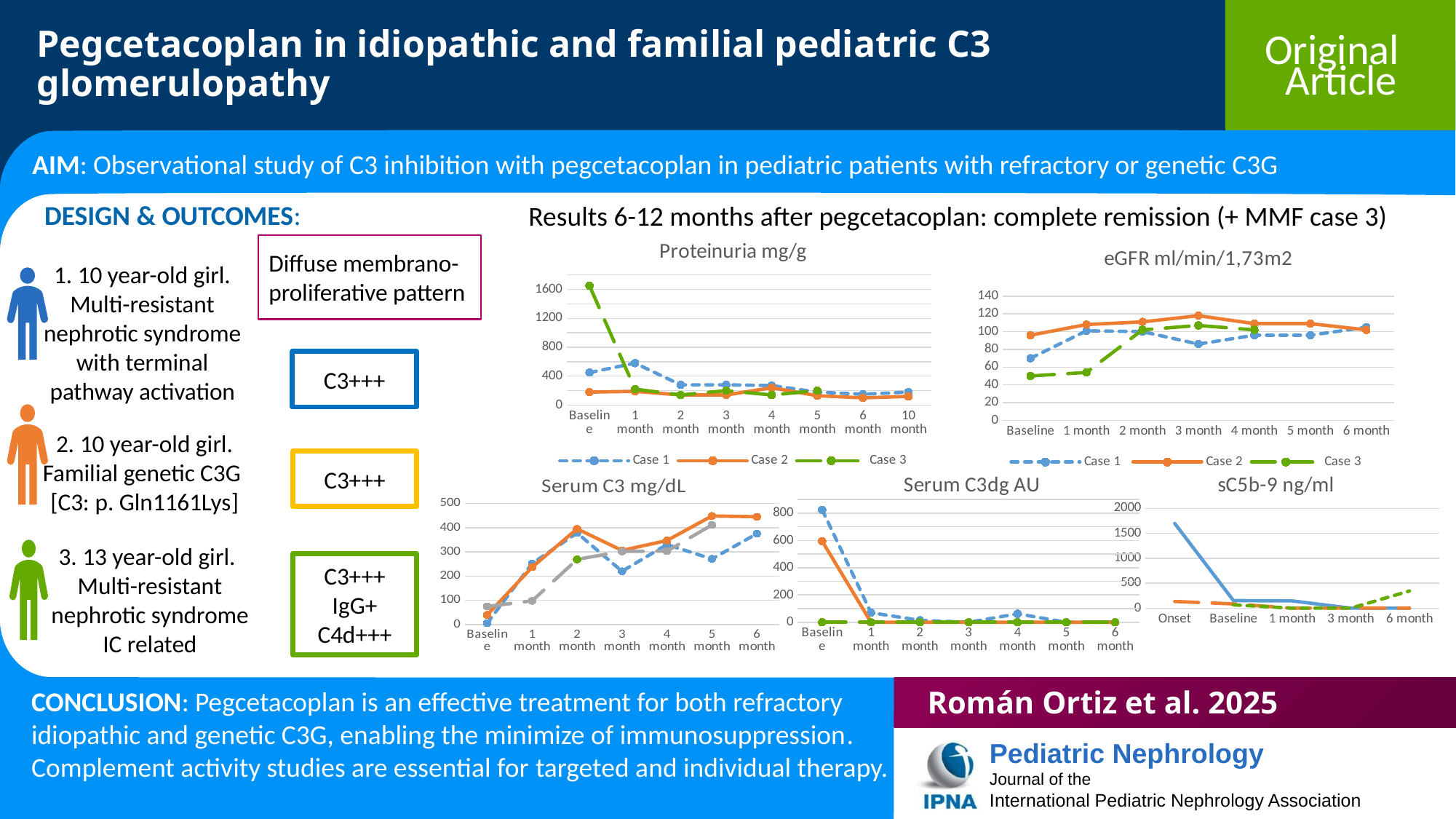

Pegcetacoplan in idiopathic and familial pediatric C3 glomerulopathy
AIM: Observational study of C3 inhibition with pegcetacoplan in pediatric patients with refractory or genetic C3G
Results 6-12 months after pegcetacoplan: complete remission (+ MMF case 3)
DESIGN & OUTCOMES:
### Chart: Proteinuria mg/g
| Category | Case 1 | Case 2 | Case 3 |
|---|---|---|---|
| Baseline | 450.0 | 180.0 | 1650.0 |
| 1 month | 580.0 | 190.0 | 220.0 |
| 2 month | 280.0 | 140.0 | 140.0 |
| 3 month | 280.0 | 140.0 | 200.0 |
| 4 month | 270.0 | 240.0 | 140.0 |
| 5 month | 180.0 | 130.0 | 200.0 |
| 6 month | 150.0 | 100.0 | None |
| 10 month | 180.0 | 120.0 | None |Diffuse membrano-proliferative pattern
### Chart: eGFR ml/min/1,73m2
| Category | Case 1 | Case 2 | Case 3 |
|---|---|---|---|
| Baseline | 70.0 | 96.0 | 50.0 |
| 1 month | 101.0 | 108.0 | 54.0 |
| 2 month | 100.0 | 111.0 | 102.0 |
| 3 month | 86.0 | 118.0 | 107.0 |
| 4 month | 96.0 | 109.0 | 102.0 |
| 5 month | 96.0 | 109.0 | None |
| 6 month | 105.0 | 102.0 | None |1. 10 year-old girl. Multi-resistant nephrotic syndrome with terminal pathway activation
C3+++
2. 10 year-old girl.
Familial genetic C3G [C3: p. Gln1161Lys]
C3+++
### Chart: Serum C3dg AU
| Category | | | |
|---|---|---|---|
| Baseline | 824.0 | 595.0 | 0.0 |
| 1 month | 70.0 | 0.0 | 0.0 |
| 2 month | 14.0 | 0.0 | 0.0 |
| 3 month | 0.0 | 0.0 | 0.0 |
| 4 month | 62.0 | 0.0 | 0.0 |
| 5 month | 0.0 | 0.0 | 0.0 |
| 6 month | 0.0 | 0.0 | 0.0 |
### Chart: sC5b-9 ng/ml
| Category | Case 1 | Case 2 | Case 3 |
|---|---|---|---|
| Onset | 1697.0 | 134.0 | None |
| Baseline | 151.0 | 85.0 | 66.0 |
| 1 month | 145.0 | 0.0 | 0.0 |
| 3 month | 0.0 | 0.0 | 0.0 |
| 6 month | 0.0 | 0.0 | 344.0 |
### Chart: Serum C3 mg/dL
| Category | | | |
|---|---|---|---|
| Baseline | 6.0 | 40.0 | 75.0 |
| 1 month | 252.0 | 237.0 | 98.0 |
| 2 month | 378.0 | 395.0 | 269.0 |
| 3 month | 220.0 | 306.0 | 302.0 |
| 4 month | 330.0 | 347.0 | 303.0 |
| 5 month | 271.0 | 448.0 | 411.0 |
| 6 month | 375.0 | 445.0 | None |3. 13 year-old girl.
Multi-resistant nephrotic syndrome IC related
C3+++
IgG+
C4d+++
Román Ortiz et al. 2025
CONCLUSION: Pegcetacoplan is an effective treatment for both refractory idiopathic and genetic C3G, enabling the minimize of immunosuppression. Complement activity studies are essential for targeted and individual therapy.
