# Supplementary material for: Pegcetacoplan in idiopathic and familial pediatric C3 glomerulopathy
Source: Pediatr Nephrol. 2025 Dec 8;41(5):1351–60. doi: 10.1007/s00467-025-07092-7 (PMC13009099; doi:10.1007/s00467-025-07092-7)
Supplement: Supplementary file 2 — Supplementary file2 (DOCX 21 KB) [file 467_2025_7092_MOESM2_ESM.docx]

Supplementary Tables

Table S1. Histological study of children with C3G treated with pegcetacoplan

|  | Case 1 | Case 2 | Case 3 | |
| --- | --- | --- | --- | --- |
| Membrano-proliferative pattern | Diffuse | Diffuse mesangial and focal membrano-proliferative | Diffuse | Diffuse |
| Endocapillary hproliferation | no | no | focal |  |
| Glomerular crescents | no | Bowman attachments 2/52 | no |  |
| Glomeruloesclerosis | no | no | no |  |
| Intersticial fibrosis/tubular atrophy | no | <5% | no | no |
| Electron microscopy | Subendothelial and paramesangial deposits | Mesangial, paramensagial, endocapillary and subendotelial deposits | - | Mesangial and subendo-thelial deposits |
| Immunofluorescence | C3 +++ | C3 +++ | IgG+++  C3 +++ | C3 +++  IgG + |
| Immunohistochemistry | - | C4d + | C4d +++ | C4d +++ |

Table S2. Complement analyses at diagnosis of C3 Glomerulonephritis

|  | Case 1 | Case 2 | Case 3 |
| --- | --- | --- | --- |
| C3 (68-188 mg/dL) | 4 | 50 | 75 |
| C4 | 34 | 24 | 23 |
| sC5b-9 (>118ng/mL) | 1697 | 134 | 66 |
| FH (90-285µg/mL) | 151 | - | 139 |
| FI (20-40 µg/mL) | 21 | - | 17 |
| Anti FH autoantibodies | Negative | Negative | Negative |
| Nephritic factors | Negative | Negative | Negative |
| Copy Number Variations  CFH-CFHRs | NO | NO | Deletion CFHR3-CFHR1 heterozygosis |
| Complement pathogenic variants. | NO | C3:p.Gln1161Lys  heterozygosis | NO |
